# Consultation with Homœpaths and the Use of Their Medicines

**Published:** 1878-10

**Authors:** 


Consultation with Homœopaths and the use of their Medicines.—We publish herewith a note from Dr. Geo. E. Robertson, of Plattesville, Wis. It is scarcely necessary to remark in connection with it that the doctor's righteous indignation against a false system has prevented him from fully appreciating the remarks made on the subject by us:

"It is very astonishing and somewhat annoying to see intelligent, well educated physicians, who must see the vast strides medical science has been making for centuries, from the investigations of the most gigantic minds in all nations, pandering to an absurdity greater than witchcraft —a so-called system of medicine, promulgated by a visionary, disappointed individual, who fell a victim to his theory; a system that would abnegate the whole medical science, and leave it in a worse condition than Hipocrates found it thousand of years ago.

"A very learned physician has said, 'if a regular physician of the old school advocates homœopathy, he is either a fool or a knave.' I can see no possible objection in our consulting with eclectics. They have no theory differing from ours; they repudiate a few drugs, and think that they have substitutes less dangerous to produce the same effect. We really owe them thanks for their investigation of many medicinal plants we all admit to be valuable. The Thomsonians, with all their ignorance, occasionally did good with a great deal of harm; but they were honest enough to practice what they believed, and the whole system has passed away like a vapor. Hydropathy, in intelligent physicians' hands, in some cases is an admirable remedy. If an ignorant negro, a Hottentot, or an Indian was to satisfy my judgment that he possessed an efficient remedy, I would use it. But the doctrine of Hahnemann has no more to sustain it than witchcraft, or the touch of the seventh son. You state that 'not one in a thousand adheres to the dogma of Hahnemann.' I admit it. Then they are dishonest, and unfit to consult with; and their medicines, by Hahnemann and his followers' stand-point would avail nothing. Consequently nothing could be gained from either consultation or medicines. If they claim to be homœopaths and practice allopathy, is it not evident that their object is to mislead the people? The honest, intelligent physician needs no such double dealing. If his patient needs no medicine, he will give none; if he needs small doses, he will give them; if his case requires heroic remedies, he will use them. If the homœopaths have seen the ridiculousness of their doctrine, and wish to be recognized as medical men, let them comply with the requisitions, and we will admit them."

